# Health Tract, No. 218

**Published:** 1864-09

**Authors:** 


### HEALTH TRACT, NO. 218.

### DIGESTIBILITY OF FOOD.

THE following table of the digestibility of the most common articles of food prepared from standard authorities, is approximately correct and is of very general practical interest:

| *Quality.* | *Preparation.* | *Time of Digestion.* H. M. |
|---|---|---|
| Rice, | Boiled, | 1 00 |
| Pigs' feet, soused, | “ | 1 00 |
| Tripe, soused, | “ | 1 00 |
| Eggs, whipped, | Raw, | 1 30 |
| Trout, salmon, fresh, | Boiled, | 1 30 |
| Trout, salmon, fresh, | Fried, | 1 30 |
| Soup, barley, | Boiled, | 1 30 |
| Apples, sweet, mellow, | Raw, | 1 30 |
| Venison steak, | Broiled, | 1 35 |
| Brains, animal, | Boiled, | 1 45 |
| Sago, | “ | 1 45 |
| Tapioca, | “ | 2 00 |
| Barley, | “ | 2 00 |
| Milk, | “ | 2 00 |
| Liver, beef's, fresh, | Broiled, | 2 00 |
| Eggs, fresh, | Raw, | 2 00 |
| Codfish, cured dry, | Boiled, | 2 00 |
| Apples, sour, mellow, | Raw, | 2 00 |
| Cabbage, with vinegar, | “ | 2 00 |
| Milk, | “ | 2 15 |
| Eggs, fresh, | Roasted, | 2 15 |
| Turkey, wild, | “ | 2 18 |
| Turkey, domestic, | Boiled, | 2 25 |
| Gelatine, | “ | 2 30 |
| Turkey, domestic, | Roasted, | 2 30 |
| Goose, wild, | “ | 2 30 |
| Pig, sucking, | “ | 2 30 |
| Lamb, fresh, | Broiled, | 2 30 |
| Hash, meat, and vegetables, | Warmed, | 2 30 |
| Beans, pod, | Boiled, | 2 30 |
| Cake, sponge, | Baked, | 2 30 |
| Parsnips, | Boiled, | 2 30 |
| Potatoes, Irish, | Roasted, | 2 30 |
| Cabbage, head, | Raw, | 2 30 |
| Spinal marrow, animal, | Boiled, | 2 40 |
| Chicken, full grown, | Fricasseed, | 2 45 |
| Custard, | Baked, | 2 45 |
| Beef, with salt only, | Boiled, | 2 45 |
| Apples, sour, hard, | Raw, | 2 50 |
| Oysters, fresh, | “ | 2 55 |
| Eggs, fresh, | Soft boiled, | 3 00 |
| Bass, striped, fresh, | Broiled, | 3 00 |
| Beef, fresh, lean, rare, | Roasted, | 3 00 |
| Pork, recently salted, | Stewed, | 3 00 |
| Mutton, fresh, | Broiled, | 3 00 |
| Soup, | Boiled, | 3 00 |
| Chicken soup, | “ | 3 00 |
| Aponeurosis, | “ | 3 00 |
| Dumpling, apple, | “ | 3 00 |
| Cake, corn, | Baked, | 3 00 |
| Oysters, fresh, | Roasted, | 3 15 |
| Porksteak, | Broiled, | 3 15 |
| Mutton, fresh, | Roasted, | 3 15 |
| Bread, corn, | Baked, | 3 15 |
| Carrot, orange, | Boiled, | 3 15 |
| Sausage, fresh, | Broiled, | 3 30 |
| Flounder, fresh, | Fried, | 3 30 |
| Catfish, fresh, | “ | 3 30 |
| Oysters, fresh, | Stewed, | 3 30 |
| Butter, | Melted, | 3 30 |
| Cheese, old, strong, | Raw, | 3 30 |
| Soup, mutton, | Boiled, | 3 30 |
| Oyster soup, | “ | 3 03 |
| Bread, wheat, fresh, | Baked, | 3 30 |
| Turnips, flat, | Boiled, | 3 30 |
| Potatoes, Irish, | “ | 3 30 |
| Eggs, fresh, | Hard boiled, | 3 30 |
| Green corn and beans, | Boiled, | 3 45 |
| Beets, | “ | 3 45 |
| Salmon, salted, | “ | 4 00 |
| Beef, | Fried, | 4 00 |
| Veal, fresh, | Broiled, | 4 00 |
| Fowls, domestic, | Roasted, | 4 00 |
| Soup, beef, vegetables, and bread, | Boiled, | 4 00 |
| Heart, animal, | Fried, | 4 00 |
| Beef, old, hard, salted, | Boiled, | 4 15 |
| Soup, marrow-bones, | “ | 4 15 |
| Cartilage, | “ | 4 15 |
| Pork, recently salted, | “ | 4 30 |
| Veal, fresh, | Fried, | 4 30 |
| Ducks, wild, | Roasted, | 4 30 |
| Suet, mutton, | Boiled, | 4 30 |
| Cabbage, | “ | 4 30 |
| Pork, fat and lean, | Roasted, | 5 15 |
| Tendon, | Boiled, | 5 30 |
| Suet, beef, fresh, | “ | 5 30 |

